# Effect of wheat straw biochar addition on canola growth in different soils

**DOI:** 10.1371/journal.pone.0335220

**Published:** 2025-11-05

**Authors:** Masooma Hassan, Vladimir Strezov

**Affiliations:** School of Natural Sciences, Faculty of Science and Engineering, Macquarie University NSW, Sydney, New South Wales, Australia; RMIT University, AUSTRALIA

## Abstract

Biochar has been demonstrated as a soil amendment to improve soil health and plant yield. The present study aimed at investigating the potential of wheat straw biochar on canola morphology and yield grown in different soils. The influence of biochar on soil physical and chemical properties was also assessed. A completely randomised design pot experiment was carried out in glasshouse where canola was planted in eight different soils with and without biochar treatment. Wheat straw biochar was incorporated in pots at 1% of the total soil weight. Canola was grown for 105 days after which its morphological and yield parameters were evaluated. Analysis of variance confirmed that biochar exerted a significant effect on shoot length, shoot and root dry weights, flower count and 100 seeds weight. Soil texture also affected canola growth and yield parameters with higher clay content in clay loam resulting in less yield compared to others. Biochar also led to improved leaf fresh and dry weight, shoot and root dry weight in loam with lower seeds weight. The seeds weight was the greatest in sandy clay loam, silty clay and silty clay loam which could be ascribed to pH changes, soil texture, decline in soil particle density and improved nutrient availability. Biochar also inflluenced increase in carbon, nitrogen and potassium levels which all helped in maximizing the yield.

## Introduction

Canola is an important oil seed crop and ranked second after soybean among the oil seed crops. Oil seeds are essential for diet after wheat, rice, oat, barley and sugar crops and they provide 2.5 times more energy compared to proteins and starch [1]. Oil seed crops are prone to different abiotic stresses, such as drought and salinity, which affect crop physiological and growth parameters. Drought reduces oil seed crop yield and degrades its quality by modifying enzymatic activity, and is also responsible for inducing other morphological and metabolic changes in plants [2].

Fluctuations in water availability and variable climate conditions have impacted agriculture on a global scale. Consequently, these phenomena have exacerbated the process of soil erosion, eutrophication and turbidity, threatening food security, water supply per capita and aquatic bodies [3]. The role of soil physical properties is also important for plant nutrient availability and water retention, however the nutrients and water status are reliant on a multitude of factors, such as structure of soil, its porous nature and aggregation in addition to other physical characteristics of soil [4].

Soil physical properties play a vital role in retaining soil water content and improving plant growth. Nutrient availability in plants relies on a number of factors, including soil aggregation, porosity and other soil physical characteristics [3,5]. Several studies have reported improved plant growth using biochar and compost as soil amendments by allowing increased nutrient availability in the soil and immobilising toxic chemicals [6]. The addition of biochar to plant soil system has been reported to foster microbial growth, plant growth and physiological characteristics, such as chlorophyll contents, shoot and root biomass [7,8].

Biochar is rich in organic carbon and is produced by pyrolysis of biomass at high temperature under no or limited oxygen conditions, with the resulting product reported to remain stable in soil for longer periods [9,10]. Some of the key benefits imparted by biochar to the soil system include improved soil structure, nutrient status and organic matter, enhanced water holding capacity, while maintaining and retaining soil moisture in its micropores allowing plants to access during period of low water availability [11,12]. According to a meta-analysis, when biochar is added to soil, 97% of its carbon is resistant to degradation and it can persist in soil up to a period of nearly 556 years [13,14].

Biochar also lowers the particle density which results in an increase in the pore volume. Similar findings for particle density and porosity have been reported in other studies [15,16]. The increase in porosity is attributed to the highly porous nature of biochar, which helps soils retain moisture and improve the flow of water [17].

Biochar promotes plant growth through reduction in particle density that leads to improved soil porosity. This improved soil structure enhances root proliferation and the well-established root system in response to biochar addition, escalating plant capacity to uptake water and nutrients [18,19]. Biochars, particularly derived from crop straw, poultry manure and livestock, provide plant growth nutrients directly as they are enriched in nitrogen, phosphorus, potassium, calcium, magnesium and other essential minerals.

Biochar has been explored for its potential to help canola cope with drought stress and improve physiological properties [10,20]. The combined potential of biochar and brassica napus in phytoextraction was explored by Cárdenas-Aguiar et al. [21] who applied manure biochar prepared at 450°C and 600°C in two mining soils and concluded that biochar promoted uptake of arsenic by *Brassica napus* in both soils. The role of canola as a zinc hyperaccumulator was reported by Belouchrani et al. [22] who demonstrated that canola had the capacity to germinate and achieve high biomass yield under high zinc concentrations. There are some other studies that have tested the capacity of biochar and *Brassica napus* in phytoremediation of contaminated soils with heavy metals and reported that *Brassica napus* had the potential to accumulate several heavy metals and biochar assisted in increasing plant biomass [21–23]. Although numerous studies report that biochar can improve nutrient availability and retention in soils, the responses vary across soil types. In a field trial, Zhao et al. [24] demonstrated that biochar enhanced rapeseed yield in acidic soil with reduced nitrogen level. The most recent study that explored the role of biochar in quantifying canola yield is based on its application in two soils. The study concluded that biochar increased P and K in clay and clay loam improving canola growth [25]. There is a strong requirement to explore how biochar responses vary across contrasting soil types when canola is cultivated as a test crop. This information is important for better management of agroecosystems supporting canola growth where biochar can reduce fertilizer application frequency due to its tendency to slowly release nutrients, which can contribute to greenhouse gas emission reductions. Thus, biochar can play a vital role in carbon sequestration in addition to improving crop growth and promoting climate resilient agriculture [17,26,27].

This study had following objectives; 1) determine the impact of biochar in improving soil properties across soil standard fertilizer applications, and 2) evaluate the potential of wheat straw biochar in improving canola growth and yield across eight soil types under uniform fertilizer application. The eight soils were selected to reflect on their textural differences and chemical properties as canola grows in diverse soil types in Australia, i.e., in clay and sandy soils of Western Australia, calcareous soils of South Australia and acidic soils of New South Wales [28]. The following hypotheses were tested in this study 1) biochar increases canola growth and yield compared to control treatment with same fertilizer application rate, and 2) biochar response varies among different soils depending on soil texture and chemical properties.

## Materials and methods

### Biochar preparation

Wheat straw biochar was produced by pyrolysis of crushed wheat straw in a fixed-bed reactor. Approximately 35 g of crushed wheat straw biomass was loaded in the reactor and the temperature inside the reactor was raised to 500°C at a continuous heating rate of 10°C/min. Nitrogen was used as a carrier gas and the total retention time for wheat straw inside the reactor was 1 hour. The obtained biochar was further ground and passed through 2 mm mesh [29]. The total amount of biochar produced was 11 g.

### Soil preparation

The river sand and topsoil were purchased from Australian Native Landscape supplies (ANLs) and clay was purchased from Blackwattle Pottery, NSW. A soil jar test was conducted to determine the texture of topsoil which showed loam texture [30]. Then sand and clay were mixed in different proportions with the loam to obtain seven different types of soil named loamy sand (LS), sandy loam (SL), silty loam (SiL), sandy clay loam (SCL), clay loam (CL), silty clay (SC) and silty clay loam (SiCL). The percentage of clay and sand was added to loam to produce seven different soil types according to the USDA soil manual [31]. The total quantity of each soil prepared for every pot was 550 g. Together with loam, eight soil types were used in this experiment. The soils were transported to a glasshouse, where they were air-dried and stored until the experiment was set up. The proportion of sand and clay added to loam is given in Table 1. Mixing sand and clay with loam in different proportions to create soils with different soil textures applied in this work has been previously used by Del Valle et al. [32] who studied the impact of artificial soils on microbial processes.

**Table 1:** Soil types and proportions of sand and clay added to loam.

Sr #	Soil type	Acronyms	% Loam	% sand	% Clay
1	Loamy sand	LS	20	65	15
2	Sandy loam	SL	30	60	10
3	Sandy clay loam	SCL	10	60	30
4	Clay loam	CL	40	20	40
5	Silty clay	SC	60	–	40
6	Silty clay loam	SiCL	60	12	28
7	Silty loam	SiL	60	20	20

### Experimental design

**Fig 1:**
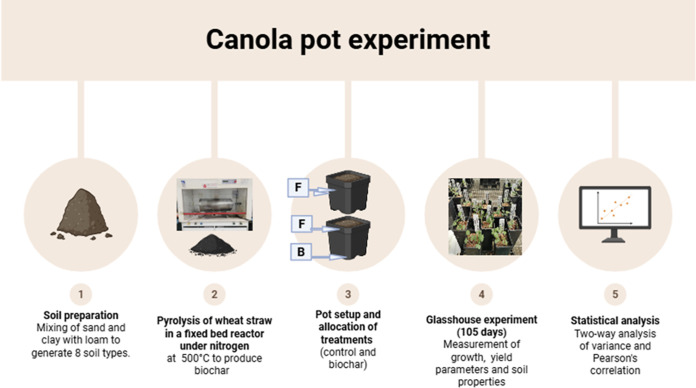
Methodology for canola pot experiment: F = fertilizer, B = biochar, Control treatment contains fertilizer, Biochar treatment includes both biochar and fertilizer (Created in https://BioRender.com).

The present study was conducted at Plant Growth Facility, School of Natural Sciences, Macquarie University. Canola seeds (*Brassica napus*) were purchased from Campsie, New South Wales (NSW), Australia. The seeds were sown in plastic pots in a completely randomised experimental design with six replicates for each treatment. The treatments were untreated soils (without biochar) and treated soils with biochar added to the pots at 1% of the total weight of soil. This application rate was selected because it was reported to be effective for enhancing crop growth and soil fertility in previous published studies [33–35]. The soil was uniformly mixed with wheat straw biochar and packed in plastic pots before sowing canola seeds. Each pot contained 550 grams of soil mixed with 1.6 g of slow-release fertiliser with NPK ratio of 16:4.4:8.3. A uniform fertiliser application rate was selected to standardise nutrient supply across all soil types, allowing testing the effect of biochar on canola yield under a fixed fertiliser rate. There were 16 treatment soil type combinations with six replicates making a total of 96 pots. The scheme of different treatments used in the study is summarised in Table 2 and the steps for canola pot experiment are illustrated in Fig 1. Additionally, images showing pots at different stages of the experiment are given in the supporting information (S1 Fig).

**Table 2:** Scheme of treatment combinations used for the experiment.

Sr #	Treatments	Composition
1	T1	LS-C
2	T2	LS-B
3	T3	SiL-C
4	T4	SiL-B
5	T5	SL-C
6	T6	SL-B
7	T7	SCL-C
8	T8	SCL-B
9	T9	CL-C
10	T10	CL-B
11	T11	SC-C
12	T12	SC-B
13	T13	SiCL-C
14	T14	SiCL-B
15	T15	Loam-C
16	T16	Loam-B

T = treatment; LS = loamy sand, SiL = silty loam, SL = sandy loam. SCL = Sandy clay loam, CL = clay loam, SC = silty clay, SiCL = Silty clay loam, C = control, B = biochar.

The temperature of the glasshouse was maintained at 26°C during the day and at 18°C during night. Plants were irrigated initially for one minute after every four hours in a day during the germination stage using the drip system. Germination was recorded on the first three days followed by thinning after 10 days with two healthy plants maintained per pot. Following this period, the water supply duration was increased to 4 minutes (4 times a day) as plants attained more height. Manual watering was then continued until the end of experiment.

### Analysis of canola growth and yield parameters

Chlorophyll content was measured using Soil Plant Analysis Development meter (SPAD meter) from intact plants [36]. For this purpose, three chlorophyll readings were taken from three different points on a leaf from three plants randomly selected from each treatment container and their average value was included in the analysis. Leaves were sampled from each treatment with leaf area meter used to determine the area of fully expanded leaves from each treatment. The other measurements included leaf fresh weight followed by dry weight taken after drying the leaves at 60°C for 72 h.

The number of flowers were also counted and the number of days for which this stage lasted was also recorded. To enhance pollen transfer, hand pollination was employed using a thin brush to facilitate transfer of pollen between vigorous flowers. The hand pollination was performed in four days interval considering that anthesis period for canola also has the same time interval [37]. The experiment continued for a period of 3.5 months after which plants were harvested and different morphological and yield related parameters were studied, including the number of pods and seeds, and the weight of 100 seeds from each canola plant.

Hundred and five days after sowing, two plants were harvested from each replicate to determine the plant growth parameters including root and shoot fresh weights, root and shoot dry weights, root and shoot length, number of leaves, leaf area, and fresh and dry leaf weight. Root and shoot dry weights were obtained by oven drying the samples at 70°C for 48 hours [38]. Yield parameters included number of pods, number of branches, number of grains and hundred seeds weight.

### Determination of soil physiochemical properties

The portable combined meter (Hannah combo pH EC meter, HI98129) was used to measure pH and electrical conductivity (EC) of biochar and soil samples. For biochar, suspension with 1:10 ratio was prepared using Milli Q water and stirred for an hour prior to collecting the data [39]. For each treatment, 550 g of air-dried soil, a subsample was drawn from which 10 g was used for the analysis. Soil suspension was prepared in 1:5 ratio using Milli Q water, stirred for 30 minutes to settle before collecting the pH reading [40]. All measurements were obtained in triplicates. C and N content were measured by conducting micro elemental analysis of the soil samples using Elementar vario MICRO (Elementar Analysensysteme GmbH) analyser [41]. Dried soil sample (2 mg) was loaded into a tin foil crucible with 5 mg of WO3 oxidation catalyst. The sample was combusted at 1150ºC with oxygen supplied at the flow rate of 30 mL/min. Sulfanilamide was used as a standard and data was recorded every 15–20 min using TCD detector. Total P and K were measured using Olympus VMR XRF, 50 KV workstation [42]. The particle density of wheat straw biomass, biochar, control, pre and post-harvest soil samples were measured using pycnometer (Micromeritics Accu Pyc II 1340). Chemical properties of the soil and biochar used for the pot experiments are shown in Table 3.

**Table 3:** Chemical properties of soil, wheat straw and biochar used prior to cultivation.

Properties	Units	Soil types								Biomass	Biochar
		Loam	LS	SL	SiL	SCL	CL	SC	SiCL	WS	WSB
**pH**		8.2	7.67	8.4	6.03	8.3	8.01	8.2	7.9	7.51	10.35
**EC**	μS/cm	527.4	192.2	264.4	130.2	263.2	114.4	97.2	105.4	347.6	1082
**Total C**	%	3.5	1.72	1.20	1.22	1.38	1.07	1.44	1.08	46.16	73.01
**Total N**	%	0.16	0.13	0.10	0.11	0.10	0.12	0.12	0.12	1.49	2.73
**Total P**	%	0.39	0.11	0.14	0.16	0.11	0.11	0.09	0.12	0.28	0.21
**Total K**	%	0.57	1.42	0.89	0.78	1.09	1.02	0.74	0.68	2.15	6.22
**Particle density**	g/cm^3^	2.43	2.61	2.58	2.61	2.63	2.66	2.64	2.60	1.46	1.61

LS = loamy sand, SL = sandy loam, SiL = Silty loam, CL = Clay loam, SCL = Sandy Clay loam, SC = Silty Clay, SiCL = Silty Clay loam; WS = wheat straw, WSB = wheat straw biochar.

### Statistical analysis

The data were analysed statistically using Minitab software (SAS Inc, Cary, NC). Data was assessed using Anderson-Darling Normality Test and all variables confirmed the assumption for normal distribution (Sig. > 0.05). Two-way ANOVA was then applied, and Tukey’s post hoc test was used for mean comparison. Pearson’s correlation was applied on normally distributed variables.

Each treatment for the canola growth and yield parameters had six replicates, while for the leaf parameters, analysis was performed on triplicates. Pearson’s correlation was also carried out to determine relationship between soil parameters, canola morphological, growth and yield parameters. The number of replicates was determined based on CSIRO plantation guidelines [43] and published studies on canola [25] and lettuce [44]. Six replicates were used for growth and yield parameters, as these are whole-plant destructive traits that typically exhibit greater biological variation, whereas leaf parameters were assessed using three replicates because they are non-destructive, repeated measurements that generally show less variation.

## Results and discussion

Table 3 summarises the chemical properties of soil, biomass and biochar determined before cultivation of canola. All the soils had alkaline pH, except SiL. The biochar used in the soil for pot experiment also had an alkaline pH and high EC.

The changes in pH, EC and other parameters after mixing the sand and clay can be associated to the differences in cation exchange capacity, stabilising organic matter and nutrient sorption of the clay and sand. Clay minerals promote strong adsorption of C, N and P, while sand promotes leaching. Previous study in which adding clay to sandy soil found increased microbial C and resulted in stablisation of organic matter [45].

### Effect on soil properties

**Fig 2:**
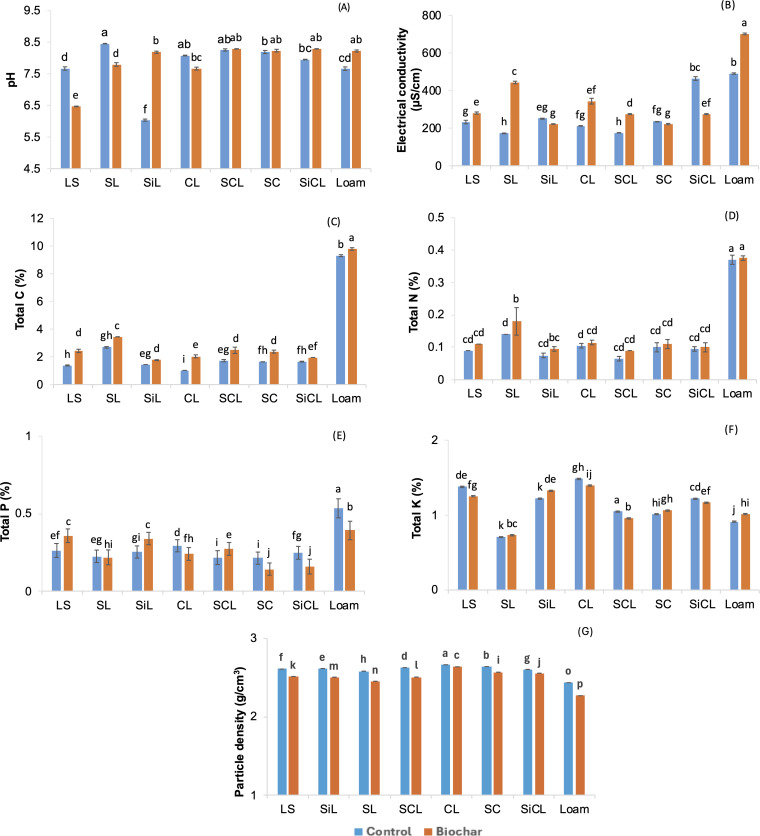
Effect of biochar on soil properties. Mean ± SD are given. Lowercase letters refer to Tukey’s groupings where means that do not share a lowercase letter are significantly different. LS = loamy sand, SL = sandy loam, SiL = silty loam, CL = clay loam, SCL = sandy clay loam, SC = silty clay, SiCL = silty clay loam.

Loamy sand (control) had a slightly alkaline pH, whereas in biochar treated loamy sand, the pH dropped to the acidic range, as shown in Fig 1A. Sandy loam control had the highest pH while it dropped in the biochar treated pot. The findings are concurrent with the previous two studies where biochar changed the pH of soils from alkaline to acidic [46,47]. This decline in pH of sandy soils occurs due to their lower buffering capacity, where oxidation of biochar and consequent production of organic acids leads to reduced pH in these soils The magnitude of biochar effect is also dependent on soil texture and even small textural modifications or changes in organic matter and cation exchange capacity can alter the degree of biochar impact [48]. Change in pH by biochar also depends on feedstock and pyrolysis conditions. Another contributing factor can be the abundance in soil microorganisms that produce acid and decrease soil pH [49]. Biochar also influenced significant increase in pH of loam, silty clay loam and silty loam compared to control. The interaction between soil type and treatments had statistically significant impact, as shown in Fig 2A.

EC was higher in biochar treated loamy sand, sandy loam, clay loam, sandy clay loam and loam. Control soils with high EC included silty loam, silty clay and silty clay loam. The interaction between treatments and soil type had a positive significant effect on EC with different letters grouping indicating significant difference, as shown in Fig 2B. Biochar reduced EC in silty loam soil by 12.6%, 5.46% in silty clay and 40.8% in silty clay loam. Decline in EC after treatment with biochar was also reported in other studies [50–52]. A study on canola by Hossain et al. [25] reported decline in pH and increase in EC in response to biochar and fertilizer addition. The increase in EC occurs due to biochar containing salts and minerals which amplify concentrations of ions in the soil solution, while decrease in EC can occur due to the adsorption or retention of nutrient ions on biochar surface or immobilisation within biochar pores [53]. The rise in EC in response to biochar addition was also reported by Hossain et al. [25] who investigated the impact of nutrient enriched biochar on soil properties and canola yield.

Soil carbon was also significantly impacted by biochar treatment, soil type and interaction between treatment and soil type (Table 4). Biochar raised soil total carbon levels in loamy sand by 75.3%, sandy clay loam (43.35%) and sandy loam (28.62%), as shown in Fig 2C. These soils were initially low in carbon, but the higher carbon content of biochar led to improved concentrations in all soil types. Similar findings on increased C content of the soils following biochar addition were highlighted in previous studies [54,55]. Biochar also led to increased carbon level in loam, but the increase was 4.9% compared to control.

**Table 4:** p-value from ANOVA test on soil parameters.

Parameters	pH	EC (μScm^-1)^)	Total carbon (%)	Total N (%)	Total P (%)	Total K (%)
**Treatment (Trt)**	0.019*	0.000***	0.000***	0.003**	0.000***	0.336^ns^
**Soil type**	0.000***	0.000***	0.000***	0.000***	0.000***	0.000***
Trt*soil type	0.000***	0.000***	0.000***	0.000***	0.000***	0.000***

Note: trt = treatment, ns = non-significant, * = significant at α = 5%, ** = significant at α = 1%, *** = significant at α = 10%.

The positive trend of biochar treatment was also found with total nitrogen, with the rate of increase comparatively lower than in case of total carbon. Individually, both biochar and soil type had positive effect on nitrogen levels, but their interaction effect was found to be non-significant, as shown in Fig 2D. The most significant increase in total nitrogen was observed in the biochar-amended sandy loam soil. Biochar can temporarily immobilise nitrogen within its porous structure, minimising losses and enabling gradual release for plant uptake. This contributes to better nitrogen retention and may lessen the need for external N [56]. The findings of the present study are in agreement with the increase in total nitrogen reported in another study [57].

A statistically significant increase in phosphorus level occurred due to the addition of biochar in loamy sand, sandy loam and sandy clay loam, Fig 2E. P was higher in the rest of the control soils where loam exhibited the highest concentration, followed by clay loam, silty clay loam and silty clay. Literature shows examples where biochar tended to decrease P concentration by serving as a sorptive medium for P [58], while elevating P levels by increasing soil pH and thus its solubility [58,59]. The positive effect of biochar combined with fertilizer on canola growth was also stated by [60,61]. Biochar can affect the available N and P pools through nutrient immobilisation by microbes, mineralisation, sorption or precipitation mechanisms [17]. Likewise, soil texture, cation exchange capacity and organic matter can regulate N and P availability. The changes in total N and P in the soil can be attributed to active interaction of biochar particles with soil particles.

Potassium levels were significantly impacted by the soil type and interaction between biochar and soil type. The highest change in K level was induced by biochar in loam followed by silty loam and silty clay, Fig 2F. The individual positive effect of biochar treatment on K^+^ concentration was also reported in a study on legumes planted in sandy soil, whereas biochar and its interaction with the second factor (year) had no significant outcome [62]. Significant increase in K^+^ in response to biochar was also reported for maize [63]. Similar results highlighting the positive influence of biochar on soil K^+^ were reported in previous studies [64,65].

Treatments, soil type and their interaction were significant for the particle density at α = 5%. Biochar caused the most prominent decline in particle density of loam, silty clay loam and silty clay. Tukey’s test confirmed the significant difference between the control and biochar treated soils by assigning the soils with different letters, as shown in Fig 2G. The lower particle density of wheat straw biochar (1.6102 g/cm^3^) and its porous nature reduced particle density in soils creating a suitable environment for water and nutrient flow. The decline in particle density and consequent improvement in some growth and soil parameters is indicated by inverse relationship in the correlation table (Table 5). The results of the present study are in agreement with two other studies which reported decline in particle density of the soils as a result of biochar application [66,67]. The addition of the biochar increases the volume of treated soils and reduces particle density which may occur due to the rearrangement of soil and biochar particles. These results are consistent with other soil–biochar incubation studies [68,69].The pressure releasing form soil-organic particles when biochar is added to soils leads to rearrangement of particles and reduction of particle density [68].

**Table 5:** Correlation matrix of soil properties and canola growth and yield parameters.

Parameters	Ger (%)	Leaf area (m^2^)	LFW (g)		LDW (g)		Chlorophyll		Shoot length (cm)		RL (cm)		SDW (g)	RDW (g)	Branches
**Ger (%)**	1														
**Leaf area (m**^**2**^)	−0.083	1													
	0.577														
**LFW (g)**	−0.444	0.311	1												
	**0.002**	**0.031**													
**LDW (g)**	−0.364	0.308	0.634		1										
	**0.011**	**0.033**	**0**												
**Chlorophyll**	−0.013	0.315	0.047		−0.06		1								
	0.931	**0.029**	0.75		0.684										
**Shoot length (cm)**	0.128	−0.246	−0.399		−0.237		−0.045		1						
	0.384	0.091	**0.005**		0.105		0.76								
**RL (cm)**	0.002	0.096	−0.071		−0.003		0.179		0.018		1				
	0.988	0.515	0.632		0.986		0.224		0.905						
**SDW (g)**	−0.065	0.196	0.173		0.152		−0.108		0.386		−0.158		1		
	0.663	0.181	0.24		0.303		0.467		**0.007**		0.283				
**RDW (g)**	−0.148	0.37	0.497		0.258		0.063		−0.029		0.068		0.569	1	
	0.316	**0.01**	**0**		0.076		0.672		0.846		0.644		**0**		
**Branches**	0.25	0.269	−0.307		−0.08		0.144		0.001		0.032		0.064	0.079	1
	0.087	0.064	**0.034**		0.59		0.327		0.992		0.828		0.666	0.594	
**Flower count**	0.306	0.363	−0.112		0.064		0.041		0.012		0.012		0.362	0.298	0.788
	**0.035**	**0.011**	0.45		0.667		0.78		0.936		0.935		**0.011**	**0.04**	**0**
**Pods**	−0.019	0.264	−0.117		0.184		−0.094		0.181		−0.119		0.288	−0.012	0.211
	0.089	0.07	0.427		0.21		0.526		0.219		0.42		**0.047**	0.937	0.151
**Seeds per pod**	−0.033	0.088	0.222		0.3		0.114		−0.133		−0.042		−0.099	0.096	−0.049
	0.824	0.554	0.129		**0.039**		0.442		0.366		0.775		0.503	0.515	0.742
**seeds weight (g)**	0.289	−0.135	−0.143		−0.101		0.259		0.047		0.102		−0.038	−0.13	0.25
	0.047	0.36	0.333		0.495		0.075		0.751		0.489		0.796	0.377	0.086
**pH**	0	0.267	0.109		0.178		−0.079		−0.374		0.16		0.041	0.188	0.138
	0.998	0.067	0.462		0.227		0.596		**0.009**		0.279		0.783	0.202	0.351
**EC (µS cm**^**-1**^)	−0.48	0.291	0.697		0.426		−0.124		−0.218		−0.104		0.329	0.483	−0.362
	**0.001**	**0.045**	**0**		**0.003**		0.402		0.136		0.481		**0.023**	**0.001**	**0.012**
**Total C (%)**	−0.424	0.231	0.876		0.506		−0.108		−0.361		−0.045		0.227	0.492	−0.382
	**0.003**	0.114	**0**		**0**		0.463		0.012		0.763		0.12	**0**	**0.007**
**Total N (%)**	−0.411	0.174	0.865		0.512		−0.147		−0.338		−0.064		0.249	0.496	−0.401
	**0.004**	0.238	**0**		**0**		0.318		**0.019**		0.668		0.088	**0**	**0.005**
**Total P (ppm)**	−0.387	0.02	0.598		0.254		−0.203		−0.068		0.064		0.19	0.328	−0.489
	**0.007**	0.893	**0**		0.082		0.167		0.646		0.665		0.195	**0.023**	**0**
**Total K (ppm)**	0.28	0.134	−0.314		−0.252		−0.034		0.173		−0.039		0.006	−0.09	0.232
	0.054	0.364	**0.03**		0.084		0.82		0.238		0.793		0.97	0.545	0.112
**Particle density** **(g cm**^**-3**^)	0.207	−0.339	−0.718		−0.348		0.019		0.188		0.005		−0.26	−0.51	0.087
	0.158	**0.019**	**0**		**0.015**		0.895		**0.2**		0.972		0.074	**0**	0.556
**Parameters**	**Flower count**	**Pods**	**Seeds per** **pod**	**Seeds weight (g)**		**pH**		**EC** **(µS cm**^**-1**^)		**Total C (%)**		**Total N (%)**	**Total P (ppm)**	**Total K (ppm)**	**Particle density (g cm**^**-3**^)
**Flower count**	1														
**Pods**	0.176	1													
	0.231														
**Seeds per pod**	−0.159	0.103	1												
	0.28	0.486													
**seeds weight (g)**	0.176	−0.224	0.193	1											
	0.232	0.126	0.189												
**pH**	0.338	−0.025	0.068	0.174		1									
	**0.019**	0.865	0.644	0.237											
**EC (µS cm**^**-1**^)	−0.214	0.03	0.266	−0.419		0.001		1							
	0.143	0.838	0.068	**0.003**		0.994									
**Total C (%)**	−0.216	−0.011	0.263	−0.217		0.093		0.81		1					
	0.141	0.943	0.071	0.138		0.529		**0**							
**Total N (%)**	−0.224	−0.043	0.279	−0.193		0.128		0.799		0.987		1			
	0.126	0.77	0.054	0.188		0.385		**0**		**0**					
**Total P (ppm)**	−0.356	−0.118	0.09	−0.324		−0.209		0.641		0.779		0.784	1		
	**0.013**	0.425	0.541	**0.025**		0.154		**0**		**0**		0			
**Total K (ppm)**	0.293	0.271	−0.22	−0.092		−0.048		−0.266		−0.35		−0.387	−0.39	1	
	**0.043**	0.062	0.133	0.532		0.746		0.068		**0.015**		**0.007**	0.006		
**Particle density (g cm**^**-3**^)	−0.021	−0.053	−0.259	0.145		−0.031		−0.74		−0.816		−0.797	−0.566	0.071	1
	0.887	0.718	0.075	0.324		0.832		**0**		**0**		**0**	0	0.63	

Note: LA = leaf area, LFW = leaf fresh weight, LDW = leaf dry weight, Ger = germination, RL = Root length, SDW = shoot dry weight, RDW = root dry weight, EC = electrical conductivity, values in bold present significant p-value.

### Effect on canola parameters

**Fig 3:**
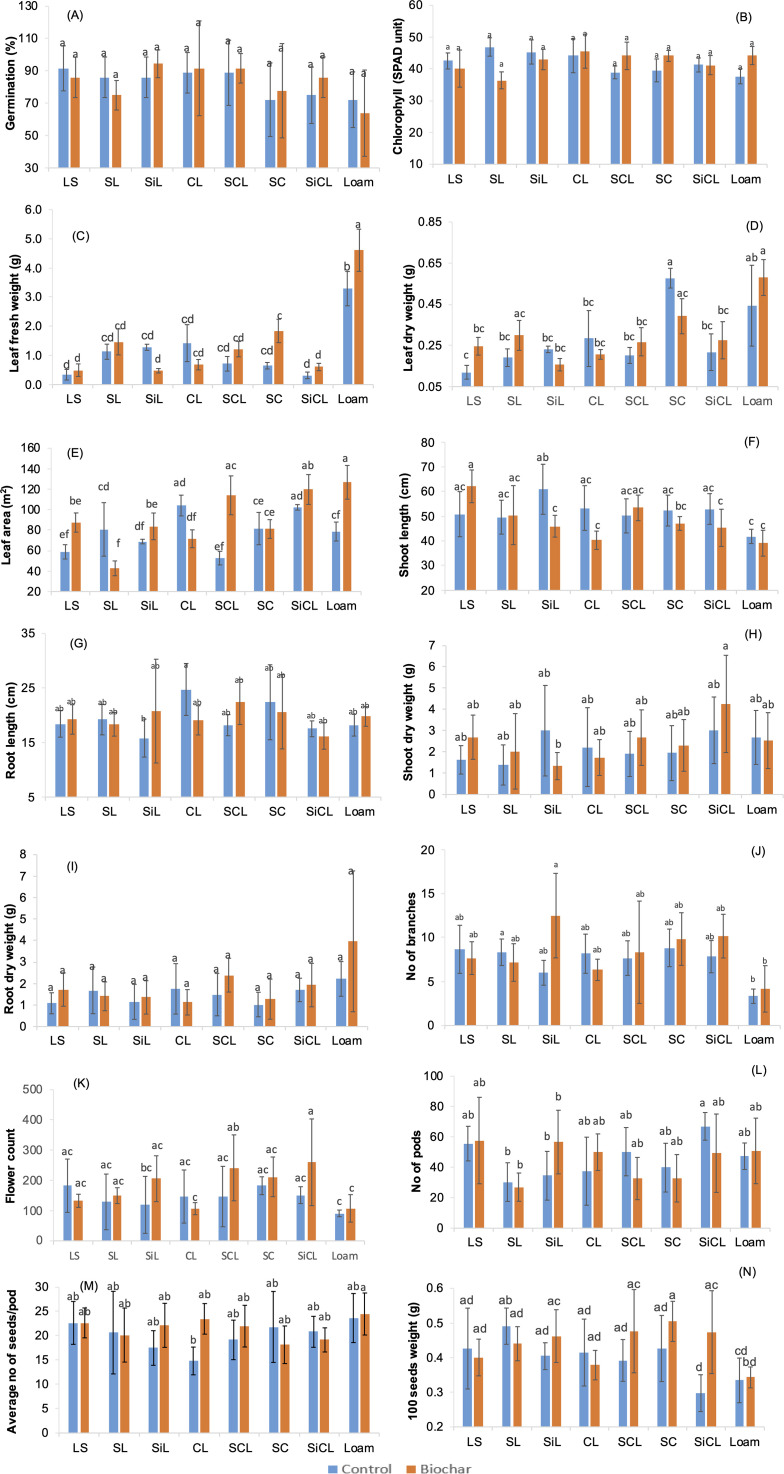
Effect of biochar and soil types on canola parameters. Mean ± SD are given. LS = loamy sand, SL = sandy loam, SiL = silty loam, CL = clay loam, SCL = sandy clay loam, SC = silty clay, SiCL = silty clay loam.. Lowercase letters refer to Tukey’s groupings where means that do not share a lowercase letter are significantly different.

Germination was recorded for a period of seven days, during which thinning was employed to maintain two healthy plants per pot. The bar graph in Fig 3A reports the germination percentage recorded for canola in each soil type and treatment. The treatment and interaction between treatment and soil type had no significant impact on germination which is evident from S1 Table and Fig 3A. The four control soil types, loamy sand, sandy loam, silty loam and loam have slightly better germination rates in contrast to the remaining soil types where biochar helped attain higher germination, but the differences were not statistically significant (S2 Table). Biochar fostered seed germination at 3.37% in clay loam and 14.56% in silty clay loam. [70] attributed the increase in seed germination rate to biochar’s higher percentage of volatile organic compounds, which stimulates several plant growth and microbial phenomena. The results of the current experiment agree with the findings from another study [71] where clay soil, because of its compact structure, showed lower emergence rate compared to silty clay and sandy loam. The decrease in germination, because of soil compaction, has been reported for different crops [72], including wheat and sorghum [73]. Clay soils can also become over logged which may cause root hypoxia and lower nutrient uptake. Thus, the higher percentage of clay can contribute to reduced germination and affect other growth parameters. Also, incomplete aging of biochar can restrict germination, reduce plant vigour, change microbial activity and availability of micronutrients through pH change and adsorption [74].

Chlorophyll is an important parameter in plants, as it, along with other pigments, such as carotenoids, play an essential role in photosynthesis by capturing sunlight. This study showed that biochar did not have a significant impact on chlorophyll content of the leaves in most treatments, as presented in Fig 3B. Only soil and treatment interaction were found to be significant (S1 Table). Clay loam, sandy clay loam, silty clay and loam amended with biochar produced canola plants with slightly more chlorophyll (3.41%, 13.3%, 11.6% and 14.7% respectively) in contrast to the controls, but these differences were not statistically significant (S2 Table; Fig 3B). Little or no improvement in chlorophyll content can be associated with short term nitrogen immobilisation, and changes in NH_4_^+^ and NO_3_^-^ pools. In addition, biochar properties and soil type can affect water retention, while reduced water flow can limit the availability of nitrogen for plant uptake [17]. The results agree with Sun et al. [63] where biochar application had no significant impact on chlorophyll content in maize. The other study in which biochar acted to enhance chlorophyll content and photosynthesis was by Khan et al. [75] who stated that the application of biochar controls soil nutrient status to sustain photosynthesis and stomatal traits, which all contributed to better yield and morphology in rapeseed.

The treatment, soil type and soil type biochar interaction all had significant impact on leaf fresh weight (S1 Table). This pronounced interaction effect is shown in Fig 3C where loam and silty clay soil were significantly influenced with the addition of biochar, with loam soil exhibiting the highest leaf fresh weight followed by silty clay. Soil type also had significant effect on leaf dry weight, α = 5% (S1 Table). Biochar significantly improved leaf area of canola in sandy clay loam by 114%, loamy sand (48.7%), silty loam (21.5%), silty clay loam (17.04%) and loam (64.6%) compared to unamended control soils, as shown in Fig 3E. These findings agree with Ullah et al. [20] who reported higher leaf area and dry weight of canola genotypes planted in biochar treated soils. Significant enhancement in leaf biomass and dry weight of spinach were also found in response to woody biochar application [76]. The better leaf dry weights and leaf area obtained in this experiment can be attributed to enhanced nitrogen availability resulting from biochar application which promotes photosynthesis and influence of soil texture on nutrient transport from roots to shoots. Loam soils provide an ideal balance of nutrients, aeration and water retention which are all conducive to the healthy growth of plant. A significant increase in leaf biomass of peanut was reported by Xu et al. [77] who showed that peanut shell biochar led to improved C and N levels in clay loam and enhanced biomass of the target crop.

Shoot length (Fig 3F), root length (Fig 3G), shoot dry weight (Fig 3H) and root dry weight (Fig 3I) were the other parameters recorded in this study. A significant increase was noted in the shoot length of canola grown in biochar treated loamy sand, while a slight rise in shoot length was observed in biochar added sandy clay loam compared to its corresponding control. In sandy loam, the shoot lengths for both treatments were similar with biochar leading to a slight positive but not significant change. Greater shoot length was observed for the plants grown in silty loam, clay loam, silty clay loam and loam control conditions. Similar result was reported in a study focused on biochar impact on pepper, where biochar promoted pepper yield, but had no impact on shoot length [78]. Among the controls, the highest shoot length for canola was recorded in silty loam while the lowest length was recorded in loam. Biochar improves porosity and water retention in sandy soils which lead to better seedling growth and reduced loss of essential nutrients. Improved aboveground biomass and yield of canola in response to nutrient enriched biochar application in clay and clay loam was reported in a study by Hossain et al. [25]. Raise in seedling parameters, such as shoot and root dry weights, has also been reported in sandy soil study, which involved testing the impact of biochar on growth of maize and black gram [70]. Increase in shoot and root biomass has also been reported by [79] who found that biochar exerted a positive impact on shoot and root growth in willow, green beans and tomatoes grown in soil containing perlite and peat moss in an ideal potting mix. The increase in shoot dry weight of green beans and tomatoes was also highlighted in other two studies conducted on clay soils [80] and sandy loam soils [80,81].

Root length did not show significant variation in control soil types, with the highest root length measured in unamended clay loam followed by silty clay. Biochar improved the root length in silty loam (31.6%), sandy clay loam (23.6%) and loam (8.1%), as shown in Fig 3G. Increased root length when biochar is added to soil is associated with the capacity of biochar to suppress ethylene concentration by adsorption onto soil. Ethylene production triggers stress in rhizosphere and inhibits root growth and microbial activity [82]. When the biochar increases root biomass, it may be due to the porous nature of biochar which lowers topsoil particle density by 10–12%, enabling the root system to develop and expand [83]. Also, biochar tends to provide more room for microbial activity to occur, which can boost soil and plant health [20].

There were slight differences in the shoot and root dry weights examined from control and biochar treated soils. However, these differences were found to be statistically insignificant. Only soil type had a significant effect on root dry weight (S1 Table). There were three control soils, silty loam, loam, clay loam and loam where shoot dry weights were higher than in the corresponding biochar treatments (S2 Table). Silty loam control plants showed the highest shoot dry weight while sandy loam controls had the lowest. For the biochar treated soils, the highest shoot dry weight was noticed in silty clay loam with 40% increase compared to its control, as presented in Fig 3H. The results are in agreement with the findings of another study where biochar increased both shoot length and shoot dry weight [84]. The possible reason for the increased shoot length and shoot dry weight in silty loam and clay loam control soils could be associated with better water holding capacity of these soils, and improved translocation of carbohydrates towards shoot compared to biochar treated soils.

The wheat straw biochar improved root dry weight in loam, sandy clay loam and loamy sand, at 45%, 54.5% and 11.7%, respectively, as shown in Fig 3I. For the rest of the soils, there were no prominent increases when biochar was applied. In control soils, silty loam and clay loam performed better than the biochar supplemented soils.

The yield parameters studied in this work were number of branches (Fig 3J), flower count (Fig 3K), number of pods (Fig 3L), number of seeds per pod (Fig 3M) and 100 seeds weight (Fig 3N). The number of branches was significantly but slightly affected by the soil type and interaction effect of biochar and soil type (S3 Table). Biochar exerted a significant impact on the number of branches in silty loam, it also led to increase in branches in sandy clay loam, silty clay loam and loam but the impact was not significant (Fig 3J). This outcome is different to the result reported by [10] on canola where acacia wood biochar significantly increased the number of branches.

Fig 3K shows that biochar increased flower count in silty loam by 73.1%, 72.1% in silty clay loam and 64.6% in sandy clay loam. Biochar also increased the flower count in loam, but the increase was not significant with both treatments assigned the same Tukey’s grouping (S4 Table). In contrast, the plausible reasons for a high flower count (Fig 3K) in loamy sand and clay loam controls compared to biochar amended soils could be related to the texture of soil, little immobilisation of nutrients in absence of biochar and better availability of nutrients for the plant uptake [85]. The number of pods exceeded the control conditions in four biochar augmented soils, namely loamy sand, silty loam, clay loam and loam. The increase in these yield parameters can occur due to biochar rich nutrient properties providing organic nitrogen, potassium and phosphorus in bioavailable forms, which are released into the soil, thus increasing the uptake of nutrients by plant roots [11].

All sources of variation had significant impact on 100 seeds weight (S3 Table). Tukey test confirmed that biochar and soil type interaction is significant for 100 seeds weight in silty loam, sandy clay loam, silty clay, silty clay loam (S4 Table). In another study on silty clay loam, biochar was also shown to promote rapeseed yield compared to control [57]. Biochar also improved seeds weight in loam, but the increase was not significant. The possible reasons for low seed weight in loam can be associated to its moderate capacity to retain nutrients, biochar induced nitrogen immobilisation and biochar soil interactions that regulate the availability of nutrients for plant consumption. Fig 3N also shows that among the controls, loamy sand, sandy loam and clay loam have comparatively higher seeds weight than the biochar treated soils, but the differences were not significant. Previous studies have confirmed that biochar improved dry weight yield of beans and tomatoes [79] grown in sandy loam. Biochar was also shown to improve canola pods, number of seeds and seeds weight when acacia wood biochar was added to sandy loam which was associated with increases in soil K^+^ and P [10]. Increased canola growth and yield in response to crop straw biochar treatment was reported in sandy clay loam [24]. In another study, it was shown that the biochars derived from wheat straw, bagasse and wood improved soybean yield in sandy loam and clay loam. [86]. Sunflower yield also improved in response to poultry litter biochar addition in sandy clay loam with increase in N, P and extractable K^+^ [87]. Canola flourishes well in sandy and clay loam soils, which are well drained and provide a favorable environment if soil compaction is also kept under control [71]. Because of their texture, these soils allow better proliferation of roots and enhance water flow, which is essential for higher yields.

The correlation matrix presented in Table 5 shows that branches, flower count and pods have a moderately positive correlation with leaf area, demonstrating that higher leaf area in canola results in increased flower count and pods. There is a strong positive correlation between flower count and branches. Table 5 also illustrates that shoot dry weight and root dry weight have a positive correlation. Phosphorus is negatively correlated with branches and flower count. A weakly positive correlation exists between leaf area and K^+^, which has previously been reported by [24]. For the other parameters, the correlation is either weakly positive or negative. It is also apparent from Table 5 that pH and phosphorus have a strong negative correlation. This could occur due to soil absorption and uptake of P by plants at alkaline pH, which reduces P concentration in soil.

Soil particle density has a strong negative correlation with leaf fresh weight and root dry weight which indicates that the decrease in particle density is associated with the increase in these parameters (Table 5). This is also evident from Fig 2G where sandy clay loam, silty clay loam and loam treated with biochar have lower particle densities and produced more leaf fresh weight and root dry weight, as shown in Fig 3 (G and I). The change in soil texture by applying biochar and the increased root length and dry weight of lettuce was also reported by [88]. The present study also reveals that particle density has a strong negative correlation with the soil nutrients C, N and P (Table 5). This is because reduction in particle density by biochar addition results in better adjustment of pore spaces and thus allows improved flow of nutrients for plant uptake. Biochar can have both beneficial and harmful effects on crop growth, as reported in meta-analyses and reviews [89,90]. This is due to the contrast in soil properties, biochar characteristics and type of crop.

Table 5 also highlights that there is a highly positive correlation between the number of branches and flower count in silty loam, sandy clay loam, silty clay and silty clay loam. The better weight of seeds in silty loam and sandy clay loam can be attributed to the high phosphorus level in soil which rose further due to the application of biochar. Phosphorus is one of the most important nutrients for plants and is closely involved in several metabolic processes. Phosphorus deficiency is one of the dominant reasons for low yields [91]. Some of the important functions that phosphorus is accounted for is its ability to stimulate root proliferation which amplifies root volume and promotes soil nutrient interactions leading to improved crop yield [92].

Biochar had a positive influence on soil properties and yield parameters but still there were negative correlations of C, N, P and K with most of the yield parameters. This higher yield is associated with more nutrient uptake by the crop which leads to low residual nutrient concentration in soil. Also, biochar can lead to immobilisation of nutrients or sorption into its porous spaces, thus limiting availability for the plant although increasing nutrient concentrations in soil.

## Conclusions

The present study assessed the impact of biochar on physical and chemical parameters of different soil types and investigated the effect of soil amendment on canola morphological and yield parameters in different soil types. Biochar exerted a positive significant effect on various parameters, like shoot length, shoot dry matter, leaf area, flower count and 100 seeds weight, which was confirmed by Tukey’s pairwise comparison test. The composition of soil also affected canola growth parameters and yield. Marked changes by addition of biochar were also noticed in soil chemical properties, such as pH, electrical conductivity, P and K content. Better seed weights were obtained from the canola grown in silty loam, silty clay, sandy clay loam and silty clay loam which was due to improvement in soil texture by biochar and immobilisation of nutrients for plant uptake. Improvement in canola leaf and growth parameters was also observed in case of loam, but the seeds harvested from this soil had lower weight. Biochar led to changes in pH from alkaline to acidic range, thus increasing the availability of P and K in these soils. Additionally, improved C and N concentrations following biochar application also tended to maximise canola yields, number of pods and seeds weight. The substantial rise in canola yields and growth parameters due to biochar application implies that wheat straw biochar can be beneficial for canola production under uniform fertilisation applications. Since these results were obtained in a pot trial experiment, further investigations at field scale and multi years can validate broader applicability of biochar. Additionally, studies should be undertaken that underscore the cost effectiveness of biochar and farmers perception to indorse adoption of biochar in sustainable agriculture.

## Supporting information

S1 Fig Canola growth stages. (DOCX)

S1 Table p-value from ANOVA test on canola growth parameters. (DOCX)

S2 Table Effect of biochar and soil type on canola growth parameters. (DOCX)

S3 Table p-value from ANOVA test on canola yield parameters. (DOCX)

S4 Table Effect of biochar and soil type on canola yield parameters. (DOCX)

## References

[1] Bukhari MA, Sharif MS, Ahmad Z, Barutçular C, Afzal M, Hossain A, et al. Silicon Mitigates the Adverse Effect of Drought in Canola (Brassica napus l.) Through Promoting the Physiological and Antioxidants Activity. Silicon. 2020;13(11):3817–26. doi: 10.1007/s12633-020-00685-x

[2] Maghsoudi K, Emam Y, Ashraf M. Foliar application of silicon at different growth stages alters growth and yield of selected wheat cultivars. Journal of Plant Nutrition. 2015;39(8):1194–203. doi: 10.1080/01904167.2015.1115876

[3] Kang MW, Yibeltal M, Kim YH, Oh SJ, Lee JC, Kwon EE, et al. Enhancement of Soil Physical Properties and Soil Water Retention Using Biochar-Based Soil Amendment. Available at SSRN 4052476. 2022. 10.1016/j.scitotenv.2022.155746 35525368

[4] Dai Y, Zheng H, Jiang Z, Xing B. Combined effects of biochar properties and soil conditions on plant growth: A meta-analysis. Sci Total Environ. 2020;713:136635. doi: 10.1016/j.scitotenv.2020.136635 32019022

[5] de Andrade Bonetti J, Anghinoni I, Ivonir Gubiani P, Cecagno D, de Moraes MT. Impact of a long-term crop-livestock system on the physical and hydraulic properties of an Oxisol. Soil and Tillage Research. 2019;186:280–91. doi: 10.1016/j.still.2018.11.003

[6] Khan AHA, Nawaz I, Yousaf S, Cheema AS, Iqbal M. Soil amendments enhanced the growth of Nicotiana alata L. and Petunia hydrida L. by stabilizing heavy metals from wastewater. J Environ Manage. 2019;242:46–55. doi: 10.1016/j.jenvman.2019.04.040 31026802

[7] Denyes MJ, Rutter A, Zeeb BA. In situ application of activated carbon and biochar to PCB-contaminated soil and the effects of mixing regime. Environ Pollut. 2013;182:201–8. doi: 10.1016/j.envpol.2013.07.016 23933124

[8] Hussain F, Hussain I, Khan AHA, Muhammad YS, Iqbal M, Soja G, et al. Combined application of biochar, compost, and bacterial consortia with Italian ryegrass enhanced phytoremediation of petroleum hydrocarbon contaminated soil. Environmental and Experimental Botany. 2018;153:80–8. doi: 10.1016/j.envexpbot.2018.05.012

[9] Major J, Lehmann J, Rondon M, Goodale C. Fate of soil‐applied black carbon: downward migration, leaching and soil respiration. Global Change Biology. 2010;16(4):1366–79. doi: 10.1111/j.1365-2486.2009.02044.x

[10] Shakeel H, Jahan S, Rafiq K, Iqbal S, Rasul F. Efficacy of Biochar-Supplemented Soil for Modification of Physio-Biochemical Attributes of Canola (Brassica napus L.) Genotypes under Different Moisture Regimes. J Soil Sci Plant Nutr. 2022;22(3):3667–84. doi: 10.1007/s42729-022-00918-5

[11] Laird D, Fleming P, Wang B, Horton R, Karlen D. Biochar impact on nutrient leaching from a Midwestern agricultural soil. Geoderma. 2010;158(3–4):436–42. doi: 10.1016/j.geoderma.2010.05.012

[12] Narzari R, Bordoloi N, Sarma B, Gogoi L, Gogoi N, Borkotoki B, et al. Fabrication of biochars obtained from valorization of biowaste and evaluation of its physicochemical properties. Bioresour Technol. 2017;242:324–8. doi: 10.1016/j.biortech.2017.04.050 28501382

[13] Shen Z, Zhang Y, Jin F, McMillan O, Al-Tabbaa A. Qualitative and quantitative characterisation of adsorption mechanisms of lead on four biochars. Sci Total Environ. 2017;609:1401–10. doi: 10.1016/j.scitotenv.2017.08.008 28797146

[14] Wang J, Xiong Z, Kuzyakov Y. Biochar stability in soil: meta‐analysis of decomposition and priming effects. GCB Bioenergy. 2015;8(3):512–23. doi: 10.1111/gcbb.12266

[15] Oladele SO. Changes in physicochemical properties and quality index of an Alfisol after three years of rice husk biochar amendment in rainfed rice – Maize cropping sequence. Geoderma. 2019;353:359–71. doi: 10.1016/j.geoderma.2019.06.038

[16] Adekiya AO, Agbede TM, Olayanju A, Ejue WS, Adekanye TA, Adenusi TT, et al. Effect of Biochar on Soil Properties, Soil Loss, and Cocoyam Yield on a Tropical Sandy Loam Alfisol. ScientificWorldJournal. 2020;2020:9391630. doi: 10.1155/2020/9391630 32158364 PMC7060867

[17] Joseph S, Cowie AL, Van Zwieten L, Bolan N, Budai A, Buss W, et al. How biochar works, and when it doesn’t: A review of mechanisms controlling soil and plant responses to biochar. GCB Bioenergy. 2021;13(11):1731–64. doi: 10.1111/gcbb.12885

[18] Liu C, Sun B, Zhang X, Liu X, Drosos M, Li L, et al. The Water-Soluble Pool in Biochar Dominates Maize Plant Growth Promotion Under Biochar Amendment. J Plant Growth Regul. 2020;40(4):1466–76. doi: 10.1007/s00344-020-10203-3

[19] Xiang Y, Deng Q, Duan H, Guo Y. Effects of biochar application on root traits: a meta‐analysis. GCB Bioenergy. 2017;9(10):1563–72. doi: 10.1111/gcbb.12449

[20] Gul-Lalay, Ullah S, Shah S, Jamal A, Saeed MF, Mihoub A, et al. Combined Effect of Biochar and Plant Growth-Promoting Rhizbacteria on Physiological Responses of Canola (Brassica napus L.) Subjected to Drought Stress. J Plant Growth Regul. 2024;43(6):1814–32. doi: 10.1007/s00344-023-11219-1

[21] Cárdenas-Aguiar E, Suárez G, Paz-Ferreiro J, Askeland MPJ, Méndez A, Gascó G. Remediation of mining soils by combining Brassica napus growth and amendment with chars from manure waste. Chemosphere. 2020;261:127798. doi: 10.1016/j.chemosphere.2020.127798 32750617

[22] Dhiman SS, Selvaraj C, Li J, Singh R, Zhao X, Kim D, et al. Phytoremediation of metal-contaminated soils by the hyperaccumulator canola (Brassica napus L.) and the use of its biomass for ethanol production. Fuel. 2016;183:107–14. doi: 10.1016/j.fuel.2016.06.025

[23] Gascó G, Álvarez ML, Paz-Ferreiro J, Méndez A. Combining phytoextraction by Brassica napus and biochar amendment for the remediation of a mining soil in Riotinto (Spain). Chemosphere. 2019;231:562–70. doi: 10.1016/j.chemosphere.2019.05.168 31151016

[24] Zhao WR, Li JY, Deng KY, Shi RY, Jiang J, Hong ZN, et al. Effects of crop straw biochars on aluminum species in soil solution as related with the growth and yield of canola (Brassica napus L.) in an acidic Ultisol under field condition. Environmental Science and Pollution Research. 2020;27:30178–89. 32451890 10.1007/s11356-020-09330-x

[25] Hossain MZ, Bahar MM, Sarkar B, Bolan N, Donne S. Fertilizer Value of Nutrient-Enriched Biochar and Response of Canola Crop. J Soil Sci Plant Nutr. 2024;24(2):2123–37. doi: 10.1007/s42729-024-01784-z

[26] Van Zwieten L, Kammann C, Cayuela ML, Singh BP, Joseph S, Kimber S, et al. Biochar effects on nitrous oxide and methane emissions from soil. Biochar for environmental management: Routledge; 2015. p. 489–520.

[27] Wang Y, Zhang W, Shang J, Shen C, Joseph SD. Chemical Aging Changed Aggregation Kinetics and Transport of Biochar Colloids. Environ Sci Technol. 2019;53(14):8136–46. doi: 10.1021/acs.est.9b00583 31185160

[28] Federation AO. Canola in Australia: 21st century progress. NSW Department of Primary Industries; 2023.

[29] Strezov V, Evans TJ, Hayman C. Thermal conversion of elephant grass (Pennisetum purpureum Schum) to bio-gas, bio-oil and charcoal. Bioresour Technol. 2008;99(17):8394–9. doi: 10.1016/j.biortech.2008.02.039 18406608

[30] Jeffers A. Soil Texture Analysis “The Jar Test”. Clemson University Home & Garden Information Center. 2018;2021.

[31] Survey USDoS. Soil survey manual. US Department of Agriculture; 1993.

[32] Del Valle I, Gao X, Ghezzehei TA, Silberg JJ, Masiello CA. Artificial Soils Reveal Individual Factor Controls on Microbial Processes. mSystems. 2022;7(4):e0030122. doi: 10.1128/msystems.00301-22 35880897 PMC9426496

[33] Gulyás M, Someus E, Klátyik S, Fuchs M, Varga ZI, Dér S, et al. Effects of Combined Application of Solid Pyrolysis Products and Digestate on Selected Soil Properties of Arenosol and Plant Growth and Composition in Laboratory Experiments. Agronomy. 2022;12(6):1440. doi: 10.3390/agronomy12061440

[34] Lehmann J, Joseph S. Biochar for environmental management: an introduction. Biochar for environmental management: Routledge; 2015. p. 1–13.

[35] Li S, Shangguan Z. Positive effects of apple branch biochar on wheat yield only appear at a low application rate, regardless of nitrogen and water conditions. J Soils Sediments. 2018;18(11):3235–43. doi: 10.1007/s11368-018-1994-3

[36] Kamran MA, Bibi S, Chen B. Preventative effect of crop straw-derived biochar on plant growth in an arsenic polluted acidic ultisol. Sci Total Environ. 2022;812:151469. doi: 10.1016/j.scitotenv.2021.151469 34742960

[37] Rosa A de S, Blochtein B, Lima DK. Honey bee contribution to canola pollination in Southern Brazil. Sci agric (Piracicaba, Braz). 2011;68(2):255–9. doi: 10.1590/s0103-90162011000200018

[38] Kul R, Arjumend T, Ekinci M, Yildirim E, Turan M, Argin S. Biochar as an organic soil conditioner for mitigating salinity stress in tomato. Soil Science and Plant Nutrition. 2021;67(6):693–706. doi: 10.1080/00380768.2021.1998924

[39] Abbas A, Naveed M, Azeem M, Yaseen M, Ullah R, Alamri S, et al. Efficiency of Wheat Straw Biochar in Combination with Compost and Biogas Slurry for Enhancing Nutritional Status and Productivity of Soil and Plant. Plants (Basel). 2020;9(11):1516. doi: 10.3390/plants9111516 33171695 PMC7695275

[40] Abujabhah IS, Bound SA, Doyle R, Bowman JP. Effects of biochar and compost amendments on soil physico-chemical properties and the total community within a temperate agricultural soil. Applied Soil Ecology. 2016;98:243–53. doi: 10.1016/j.apsoil.2015.10.021

[41] Fatima B, Bibi F, Ishtiaq Ali M, Woods J, Ahmad M, Mubashir M, et al. Accompanying effects of sewage sludge and pine needle biochar with selected organic additives on the soil and plant variables. Waste Manag. 2022;153:197–208. doi: 10.1016/j.wasman.2022.08.016 36108538

[42] Tavares TR, Minasny B, McBratney A, Cherubin MR, Marques GT, Ragagnin MM, et al. Estimating plant-available nutrients with XRF sensors: Towards a versatile analysis tool for soil condition assessment. Geoderma. 2023;439:116701. doi: 10.1016/j.geoderma.2023.116701

[43] Poorter H, Fiorani F, Stitt M, Schurr U, Finck A, Gibon Y, et al. The art of growing plants for experimental purposes: a practical guide for the plant biologist. Funct Plant Biol. 2012;39(11):821–38. doi: 10.1071/FP12028 32480833

[44] Christou A, Stylianou M, Georgiadou EC, Gedeon S, Ioannou A, Michael C, et al. Effects of biochar derived from the pyrolysis of either biosolids, manure or spent coffee grounds on the growth, physiology and quality attributes of field-grown lettuce plants. Environmental Technology & Innovation. 2022;26:102263. doi: 10.1016/j.eti.2021.102263

[45] Riaz M, Marschner P. Sandy Soil Amended with Clay Soil: Effect of Clay Soil Properties on Soil Respiration, Microbial Biomass, and Water Extractable Organic C. J Soil Sci Plant Nutr. 2020;20(4):2465–70. doi: 10.1007/s42729-020-00312-z

[46] Liu D, Feng Z, Zhu H, Yu L, Yang K, Yu S. Effects of corn straw biochar application on soybean growth and alkaline soil properties. BioResources. 2020;15(1).

[47] Zhang M, Riaz M, Zhang L, El-Desouki Z, Jiang C. Biochar Induces Changes to Basic Soil Properties and Bacterial Communities of Different Soils to Varying Degrees at 25 mm Rainfall: More Effective on Acidic Soils. Front Microbiol. 2019;10:1321. doi: 10.3389/fmicb.2019.01321 31249563 PMC6582450

[48] Joseph SD, Camps-Arbestain M, Lin Y, Munroe P, Chia CH, Hook J, et al. An investigation into the reactions of biochar in soil. Soil Research. 2010;48(7):501–15. doi: 10.1071/sr10009

[49] Kim H-S, Kim K-R, Yang JE, Ok YS, Owens G, Nehls T, et al. Effect of biochar on reclaimed tidal land soil properties and maize (Zea mays L.) response. Chemosphere. 2016;142:153–9. doi: 10.1016/j.chemosphere.2015.06.041 26138709

[50] Dahlawi S, Naeem A, Rengel Z, Naidu R. Biochar application for the remediation of salt-affected soils: Challenges and opportunities. Sci Total Environ. 2018;625:320–35. doi: 10.1016/j.scitotenv.2017.12.257 29289780

[51] Lashari MS, Ye Y, Ji H, Li L, Kibue GW, Lu H, et al. Biochar-manure compost in conjunction with pyroligneous solution alleviated salt stress and improved leaf bioactivity of maize in a saline soil from central China: a 2-year field experiment. J Sci Food Agric. 2015;95(6):1321–7. doi: 10.1002/jsfa.6825 25042565

[52] Yue Y, Guo WN, Lin QM, Li GT, Zhao XR. Improving salt leaching in a simulated saline soil column by three biochars derived from rice straw ( Oryza sativa L.), sunflower straw ( Helianthus annuus ), and cow manure. Journal of Soil and Water Conservation. 2016;71(6):467–75. doi: 10.2489/jswc.71.6.467

[53] Joseph S, Husson O, Graber E, Van Zwieten L, Taherymoosavi S, Thomas T, et al. The Electrochemical Properties of Biochars and How They Affect Soil Redox Properties and Processes. Agronomy. 2015;5(3):322–40. doi: 10.3390/agronomy5030322

[54] Lévesque V, Oelbermann M, Ziadi N. Biochar in temperate soils: opportunities and challenges. Can J Soil Sci. 2022;102(1):1–26. doi: 10.1139/cjss-2021-0047

[55] Prommer J, Wanek W, Hofhansl F, Trojan D, Offre P, Urich T, et al. Biochar decelerates soil organic nitrogen cycling but stimulates soil nitrification in a temperate arable field trial. PLoS One. 2014;9(1):e86388. doi: 10.1371/journal.pone.0086388 24497947 PMC3907405

[56] Darby I, Xu C-Y, Wallace HM, Joseph S, Pace B, Bai SH. Short-term dynamics of carbon and nitrogen using compost, compost-biochar mixture and organo-mineral biochar. Environ Sci Pollut Res Int. 2016;23(11):11267–78. doi: 10.1007/s11356-016-6336-7 26924699

[57] Jin Z, Chen C, Chen X, Hopkins I, Zhang X, Han Z, et al. The crucial factors of soil fertility and rapeseed yield - A five year field trial with biochar addition in upland red soil, China. Sci Total Environ. 2019;649:1467–80. doi: 10.1016/j.scitotenv.2018.08.412 30308915

[58] Soinne H, Keskinen R, Heikkinen J, Hyväluoma J, Uusitalo R, Peltoniemi K, et al. Are there environmental or agricultural benefits in using forest residue biochar in boreal agricultural clay soil? Sci Total Environ. 2020;731:138955. doi: 10.1016/j.scitotenv.2020.138955 32417473

[59] Saarnio S, Räty M, Hyrkäs M, Virkajärvi P. Biochar addition changed the nutrient content and runoff water quality from the top layer of a grass field during simulated snowmelt. Agriculture, Ecosystems & Environment. 2018;265:156–65. doi: 10.1016/j.agee.2018.06.007

[60] Dannhauser A, Schoenau JJ, Hangs RD, Patra BR, Dalai AK. Biochar Amendments to Improve Soil Phosphorus Fertility and Retention in Canadian Prairie Soils. J Soil Sci Plant Nutr. 2024;24(4):6707–17. doi: 10.1007/s42729-024-01998-1

[61] Keykhosravi H, Abbaspour A, Asghari HR. Effect of rice bran biochar, mixed and enriched with triple superphosphate, on the availability of phosphorus and growth of corn. Iranian Journal of Soil Research. 2020;34(3):329–41.

[62] Jeffery S, van de Voorde TFJ, Harris WE, Mommer L, Van Groenigen JW, De Deyn GB, et al. Biochar application differentially affects soil micro-, meso-macro-fauna and plant productivity within a nature restoration grassland. Soil Biology and Biochemistry. 2022;174:108789. doi: 10.1016/j.soilbio.2022.108789

[63] Sun J, Jia Q, Li Y, Zhang T, Chen J, Ren Y, et al. Effects of Arbuscular Mycorrhizal Fungi and Biochar on Growth, Nutrient Absorption, and Physiological Properties of Maize (Zea mays L.). J Fungi (Basel). 2022;8(12):1275. doi: 10.3390/jof8121275 36547608 PMC9782859

[64] He K, He G, Wang C, Zhang H, Xu Y, Wang S, et al. Biochar amendment ameliorates soil properties and promotes Miscanthus growth in a coastal saline-alkali soil. Applied Soil Ecology. 2020;155:103674. doi: 10.1016/j.apsoil.2020.103674

[65] Rafael RBA, Fernández-Marcos ML, Cocco S, Ruello ML, Fornasier F, Corti G. Benefits of Biochars and NPK Fertilizers for Soil Quality and Growth of Cowpea (Vigna unguiculata L. Walp.) in an Acid Arenosol. Pedosphere. 2019;29(3):311–33. doi: 10.1016/s1002-0160(19)60805-2

[66] Githinji L. Effect of biochar application rate on soil physical and hydraulic properties of a sandy loam. Archives of Agronomy and Soil Science. 2013;60(4):457–70. doi: 10.1080/03650340.2013.821698

[67] Usowicz B, Lipiec J, Łukowski M, Marczewski W, Usowicz J. The effect of biochar application on thermal properties and albedo of loess soil under grassland and fallow. Soil and Tillage Research. 2016;164:45–51. doi: 10.1016/j.still.2016.03.009

[68] Gamage DNV, Mapa RB, Dharmakeerthi RS, Biswas A. Effect of rice-husk biochar on selected soil properties in tropical Alfisols. Soil Research. 2016;54(3):302–10. doi: 10.1071/sr15102

[69] Jien S-H, Wang C-S. Effects of biochar on soil properties and erosion potential in a highly weathered soil. Catena. 2013;110:225–33. doi: 10.1016/j.catena.2013.06.021

[70] Das SK, Ghosh GK, Avasthe R. Ecotoxicological responses of weed biochar on seed germination and seedling growth in acidic soil. Environmental Technology & Innovation. 2020;20:101074. doi: 10.1016/j.eti.2020.101074

[71] Owusu-Sekyere E, Chen Y. The Effect of Varying Compaction Levels on Soil Dynamic Properties and the Growth of Canola (Brassica napus L.). Agriculture. 2024;14(11):1976. doi: 10.3390/agriculture14111976

[72] Buttery BR, Tan CS, Drury CF, Park SJ, Armstrong RJ, Park KY. The effects of soil compaction, soil moisture and soil type on growth and nodulation of soybean and common bean. Can J Plant Sci. 1998;78(4):571–6. doi: 10.4141/p97-132

[73] Radford BJ, Yule DF, McGarry D, Playford C. Crop responses to applied soil compaction and to compaction repair treatments. Soil and Tillage Research. 2001;61(3–4):157–66. doi: 10.1016/s0167-1987(01)00194-5

[74] Cui W, Bai Q, Liu J, Chen J, Qi Z, Zhou W. Phytotoxicity Removal Technologies for Agricultural Waste as a Growing Media Component: A Review. Agronomy. 2023;14(1):40. doi: 10.3390/agronomy14010040

[75] Khan Z, Khan MN, Zhang K, Luo T, Zhu K, Hu L. The application of biochar alleviated the adverse effects of drought on the growth, physiology, yield and quality of rapeseed through regulation of soil status and nutrients availability. Industrial Crops and Products. 2021;171:113878. doi: 10.1016/j.indcrop.2021.113878

[76] Jabborova D, Annapurna K, Paul S, Kumar S, Saad HA, Desouky S, et al. Beneficial Features of Biochar and Arbuscular Mycorrhiza for Improving Spinach Plant Growth, Root Morphological Traits, Physiological Properties, and Soil Enzymatic Activities. J Fungi (Basel). 2021;7(7):571. doi: 10.3390/jof7070571 34356950 PMC8307178

[77] Xu C-Y, Bai SH, Hao Y, Rachaputi RCN, Xu Z, Wallace HM. Peanut shell biochar improves soil properties and peanut kernel quality on a red Ferrosol. J Soils Sediments. 2015;15(11):2220–31. doi: 10.1007/s11368-015-1242-z

[78] Graber ER, Meller Harel Y, Kolton M, Cytryn E, Silber A, Rav David D, et al. Biochar impact on development and productivity of pepper and tomato grown in fertigated soilless media. Plant Soil. 2010;337(1–2):481–96. doi: 10.1007/s11104-010-0544-6

[79] Sheffield SB, Hoefer TA, Petersen JE. Biochar has positive but distinct impacts on root, shoot, and fruit production in beans, tomatoes, and willows. Front Sustain Food Syst. 2024;8. doi: 10.3389/fsufs.2024.1346529

[80] Güereña DT, Lehmann J, Thies JE, Enders A, Karanja N, Neufeldt H. Partitioning the contributions of biochar properties to enhanced biological nitrogen fixation in common bean (Phaseolus vulgaris). Biol Fertil Soils. 2015;51(4):479–91. doi: 10.1007/s00374-014-0990-z

[81] Ronga D, Caradonia F, Parisi M, Bezzi G, Parisi B, Allesina G, et al. Using Digestate and Biochar as Fertilizers to Improve Processing Tomato Production Sustainability. Agronomy. 2020;10(1):138. doi: 10.3390/agronomy10010138

[82] Abdi H, Williams LJ, Valentin D. Multiple factor analysis: principal component analysis for multitable and multiblock data sets. WIREs Computational Stats. 2013;5(2):149–79. doi: 10.1002/wics.1246

[83] Liu B, Li H, Li H, Zhang A, Rengel Z. Long‐term biochar application promotes rice productivity by regulating root dynamic development and reducing nitrogen leaching. GCB Bioenergy. 2020;13(1):257–68. doi: 10.1111/gcbb.12766

[84] Mollinedo J, Schumacher T, Chintala R. Biochar effects on phenotypic characteristics of “wild” and “sickle”. Medicago Truncatula. 2016:1–14.

[85] Laird DA, Novak JM, Collins HP, Ippolito JA, Karlen DL, Lentz RD, et al. Multi-year and multi-location soil quality and crop biomass yield responses to hardwood fast pyrolysis biochar. Geoderma. 2017;289:46–53. doi: 10.1016/j.geoderma.2016.11.025

[86] Jahan S, Iqbal S, Rasul F, Jabeen K. Efficacy of biochar as soil amendments for soybean (Glycine max L.) morphology, physiology, and yield regulation under drought. Arab J Geosci. 2020;13(10). doi: 10.1007/s12517-020-05318-6

[87] Aziz MA, Wattoo FM, Khan F, Hassan Z, Mahmood I, Anwar A, et al. Biochar and Polyhalite Fertilizers Improve Soil’s Biochemical Characteristics and Sunflower (Helianthus annuus L.) Yield. Agronomy. 2023;13(2):483. doi: 10.3390/agronomy13020483

[88] Bruun EW, Petersen CT, Hansen E, Holm JK, Hauggaard‐Nielsen H. Biochar amendment to coarse sandy subsoil improves root growth and increases water retention. Soil Use and Management. 2014;30(1):109–18. doi: 10.1111/sum.12102

[89] Jeffery S, Verheijen FGA, van der Velde M, Bastos AC. A quantitative review of the effects of biochar application to soils on crop productivity using meta-analysis. Agriculture, Ecosystems & Environment. 2011;144(1):175–87. doi: 10.1016/j.agee.2011.08.015

[90] Kavitha B, Reddy PVL, Kim B, Lee SS, Pandey SK, Kim K-H. Benefits and limitations of biochar amendment in agricultural soils: A review. J Environ Manage. 2018;227:146–54. doi: 10.1016/j.jenvman.2018.08.082 30176434

[91] Paisey EK, Santosa E, Matra DD, Kurniawati A, Supijatno S. Self-pruning in lime (<i>Citrus aurantifolia</i> Swingle) after treatments with ichiphon, abscisic acid and nitrogen, phosphorus, and potassium fertilizers. Acta Agro. 2023;76. doi: 10.5586/aa/168236

[92] Khan MR, Rahman MdH, Tarafder MdMA, Haque MdA, Hossain MdB, Islam AT, et al. Determination of critical limit of soil phosphorous for mustard (<i>Brassica napus</i> L.) and maize (<i>Zea mays</i> L.) in different agroecological zones of Bangladesh. Acta Agro. 2024;77:1–16. doi: 10.5586/aa/174958

