# Supplementary material for: Effect of wheat straw biochar addition on canola growth in different soils
Source: PLoS One. 2025 Nov 5;20(11):e0335220. doi: 10.1371/journal.pone.0335220 (PMC12588495; doi:10.1371/journal.pone.0335220)
Supplement: S1 Fig — (DOCX) [file pone.0335220.s001.docx]

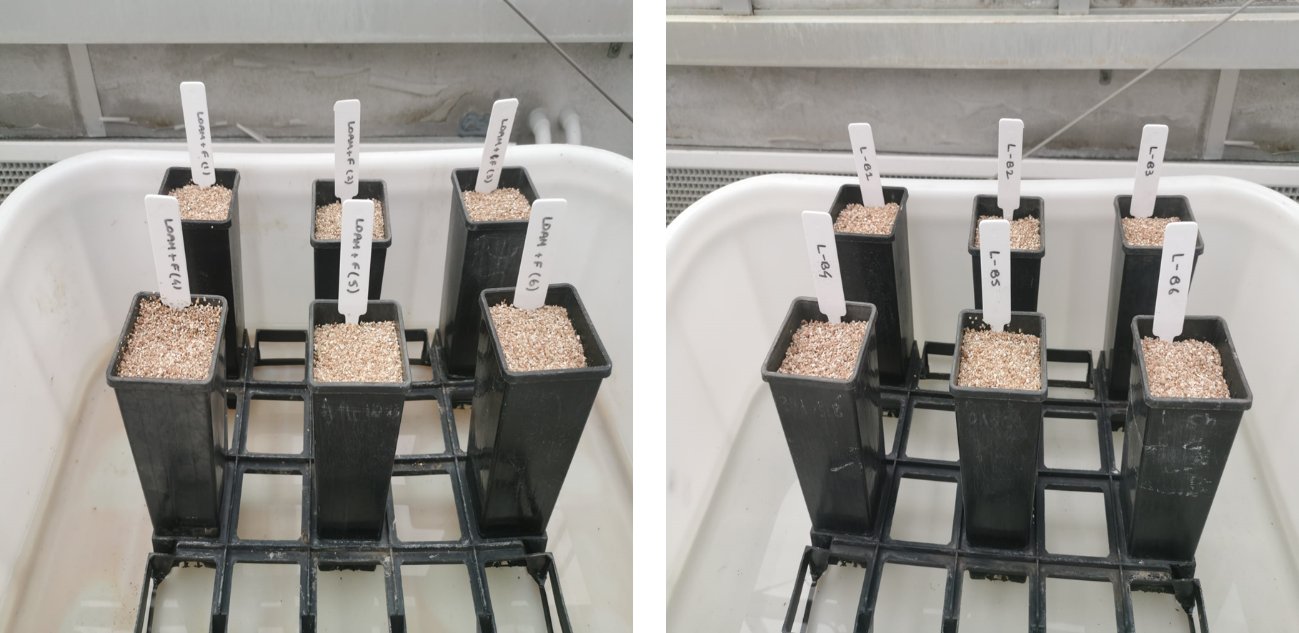


1. **Loam (control) b) Loam (biochar)**


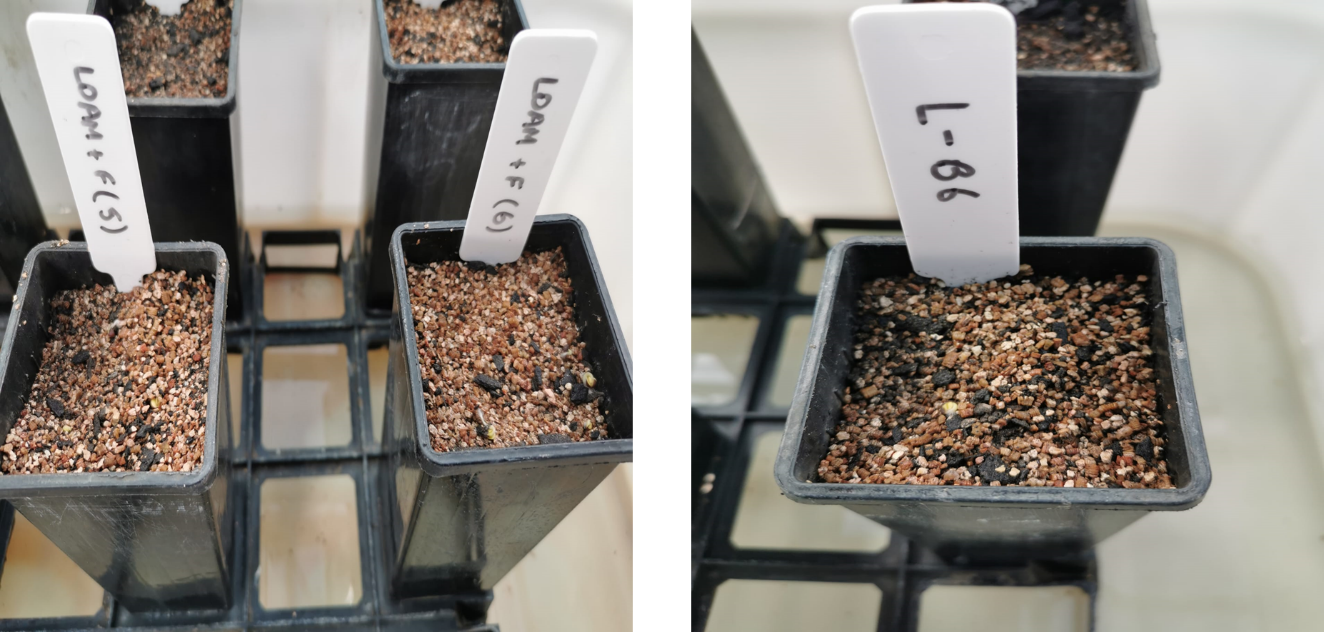


1. **Germination of canola**

**
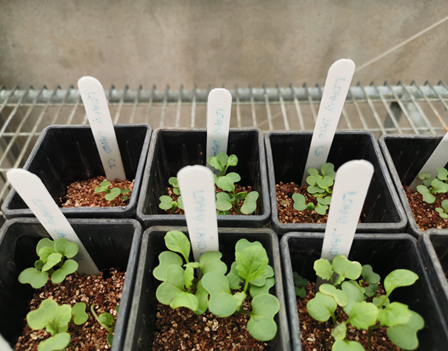
**

1. **Two leaf stage**

**
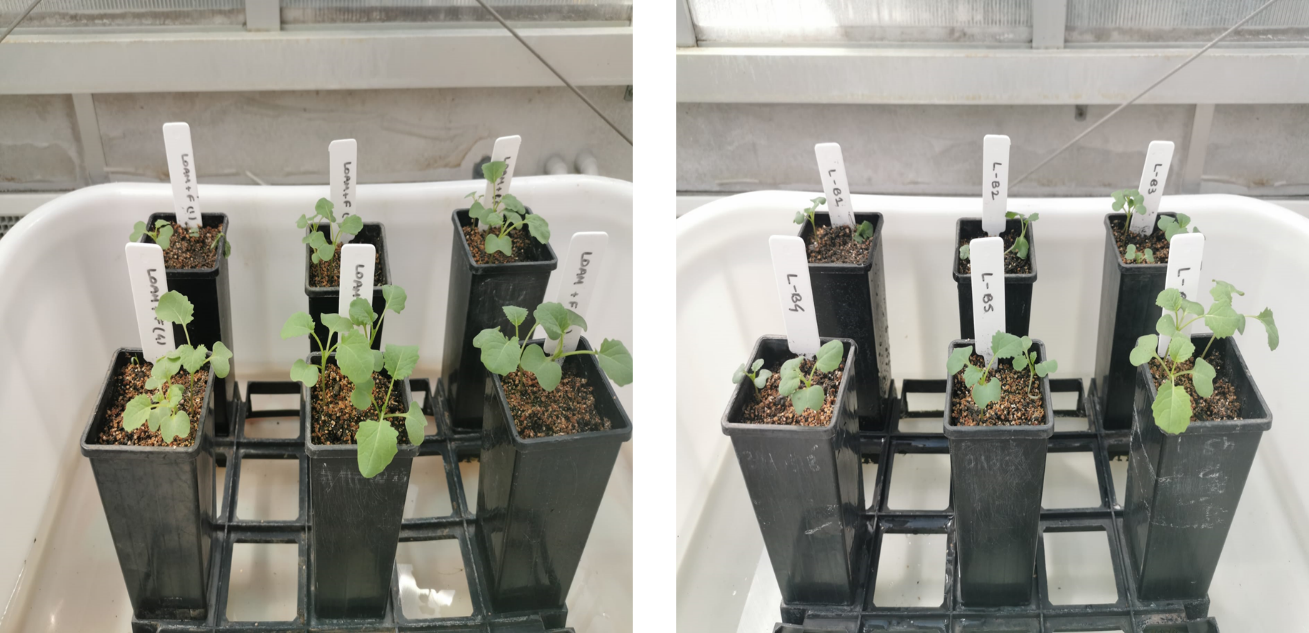
**

1. **Four leaf stage**

**S
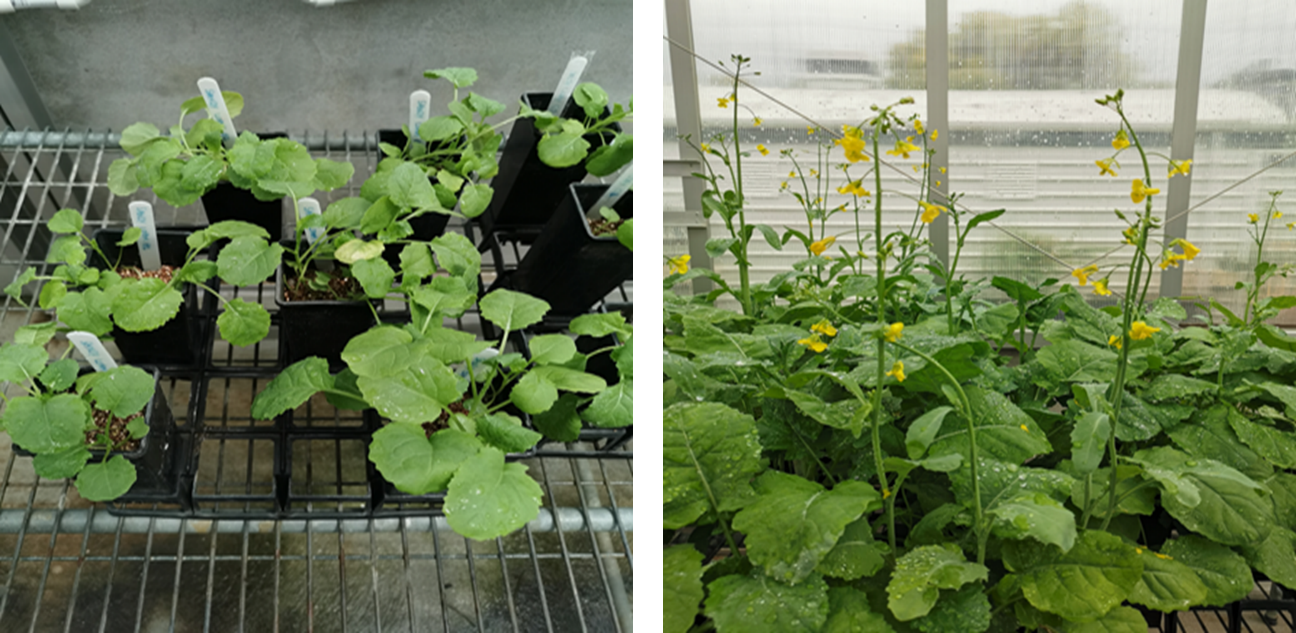
**

1. **Six leaf stage g) Flowering**

**
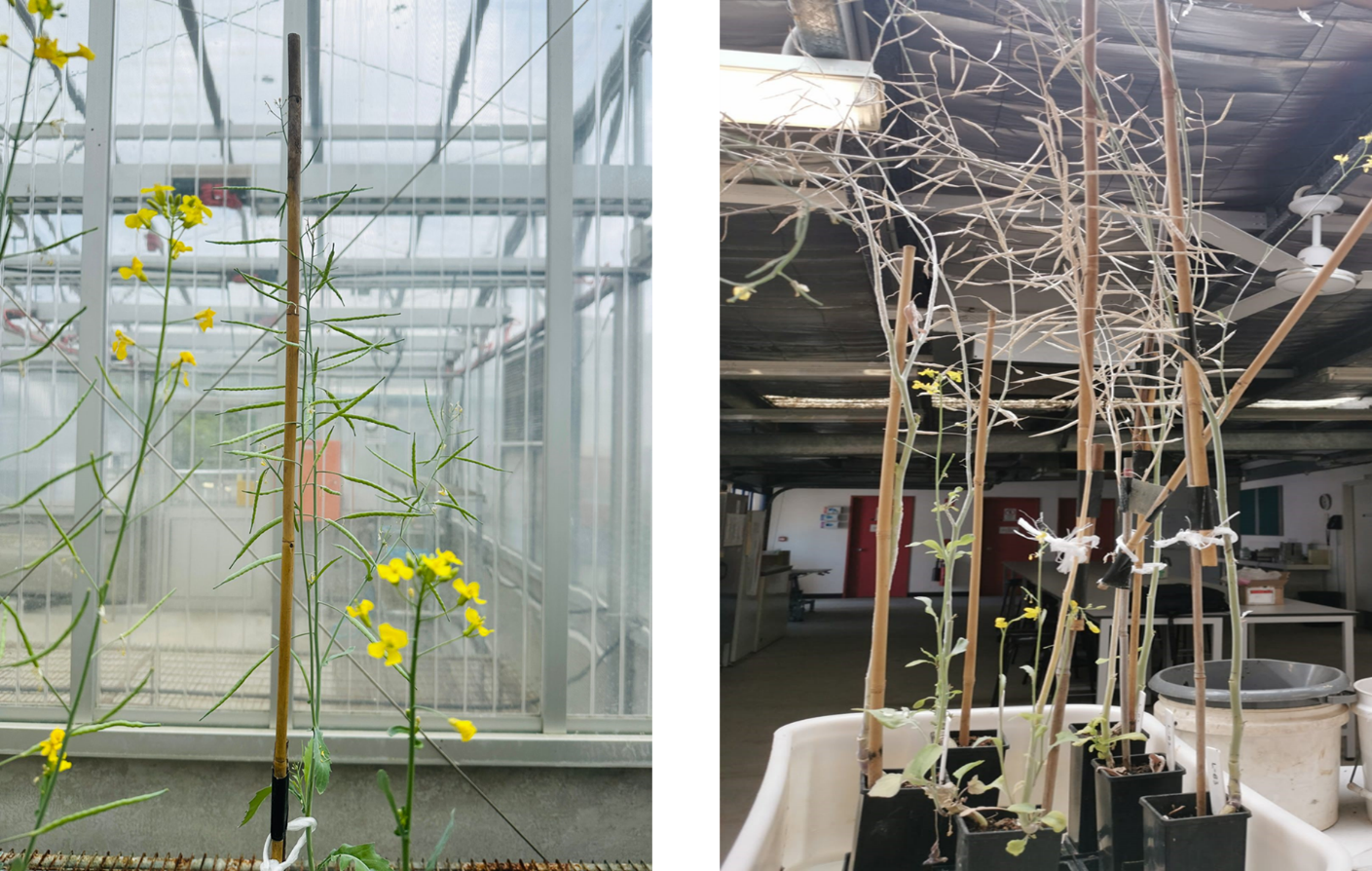
**

1. **Pod development and seed filling h) Drying (maturity stage)**

**S1 Fig. Canola growth stages**
