# Supplementary material for: Effect of wheat straw biochar addition on canola growth in different soils
Source: PLoS One. 2025 Nov 5;20(11):e0335220. doi: 10.1371/journal.pone.0335220 (PMC12588495; doi:10.1371/journal.pone.0335220)
Supplement: S1 Table — (DOCX) [file pone.0335220.s002.docx]

| **S1 Table. p-value from ANOVA test on canola growth parameters** | | | | |  |  |  |  |  |
| --- | --- | --- | --- | --- | --- | --- | --- | --- | --- |
| **Parameters** | **Germination (%)** | **LA (m^2^)** | **LFW (g)** | **LDW (g)** | **Chlorophyll** | **Shoot length (cm)** | **RL (cm)** | **SDW (g)** | **RDW (g)** |
| **Treatment (Trt)** | 0.777^ns^ | 0.002** | 0.021* | 0.474^ns^ | 0.767^ns^ | 0.016* | 0.772^ns^ | 0.443^ns^ | 0.085^ns^ |
| **Soil type** | 0.014* | 0*** | 0.000*** | 0.000* | 0.435^ns^ | 0.000*** | 0.078^ns^ | 0.056^ns^ | 0.007** |
| **Trt*soil type** | 0.728^ns^ | 0*** | 0.000*** | 0.051^ns^ | 0.006** | 0.000*** | 0.068^ns^ | 0.218^ns^ | 0.360^ns^ |

Note: trt = treatment, ns = non-significant, * = significant at α =5%, ** = significant at α = 1%, *** = significant at α = 10%.
