# Supplementary material for: Effect of wheat straw biochar addition on canola growth in different soils
Source: PLoS One. 2025 Nov 5;20(11):e0335220. doi: 10.1371/journal.pone.0335220 (PMC12588495; doi:10.1371/journal.pone.0335220)
Supplement: S2 Table — (DOCX) [file pone.0335220.s003.docx]

| **S2 Table. Effect of biochar and soil type on canola growth parameters** | | | | |  |  |  |  |  |  |
| --- | --- | --- | --- | --- | --- | --- | --- | --- | --- | --- |
| **Treatments** | | **Germination %** | **LA (m^2^)** | **LFW (g)** | **LDW (g)** | **SL (cm)** | **RL (cm)** | **SDW (g)** | **RDW (g)** | **Chlorophyll** |
| LS | Control | 91.66±13.94 a | 58.77±6.99 ef | 0.34±0.18 d | 0.12±0.03 c | 50.88±9.14 ac | 18.43±2.44 ab | 1.62±0.66 ab | 1.08±0.48 a | 42.5±2.52 a |
|  | Biochar | 86.11±12.54 a | 87.41±9.37 be | 0.496±0.21d | 0.246±0.04 bc | 62.15±6.63 a | 19.23±2.69 ab | 2.69±1.03 ab | 1.72±0.77 a | 40.1±5.83 a |
| SL | Control | 86.11±12.54 a | 80.7±26.14 cd | 1.127±0.25 cd | 0.190±0.04 bc | 49.66±6.86 ac | 19.21±2.80 ab | 1.38±0.94 ab | 1.68±1.08 a | 46.9±2.88 a |
|  | Biochar | 75±9.12 a | 42.65±7.23 f | 1.464±0.44 cd | 0.299±0.04 ac | 50.48±11.95 ac | 18.35±2.17 ab | 2.03±1.77 ab | 1.40±0.67 a | 36.4±2.66 a |
| SiL | Control | 86.11±12.54 a | 68.83±2.24 df | 1.280±0.10 cd | 0.231±0.01bc | 60.96±10.13 ab | 15.81±3.48 b | 3.00±2.12 ab | 1.15±0.81 a | 45.3±3.80 a |
|  | Biochar | 94.44±8.6 a | 83.68±13.05 be | 0.475±0.07 d | 0.156±0.03bc | 46±4.41 c | 20.76±9.51 ab | 1.33±0.63 b | 1.35± 0.77 a | 42.9± 3.22 a |
| SCL | Control | 88.88±20.18 a | 52.66±6.67 ef | 0.713±0.25 cd | 0.201±0.03bc | 50.18±6.91 ac | 18.18±1.93 ab | 1.90±1.06 ab | 1.48±0.98 a | 38.9±2.00 a |
|  | Biochar | 91.66±9.12 a | 114.00±18.99 ac | 1.216±0.26 cd | 0.268±0.06 bc | 53.41±5.18 ac | 22.45±4.14 ab | 2.67±1.30 ab | 2.39±0.78 a | 44.1±4.32 a |
| CL | Control | 88.88±12.54 a | 104.04±10.15 ad | 1.421±0.63 cd | 0.283±0.13 bc | 53.36±9.06 ac | 24.73±4.75 a | 2.23±1.85 ab | 1.75±1.17 a | 44.1±5.32 a |
|  | Biochar | 91.66±9.12 a | 71.55±8.74 df | 0.682±0.17 cd | 0.206±0.02 bc | 40.3±3.71 c | 19.06±2.67 ab | 1.73±0.84 ab | 1.12±0.59 a | 45.6±5.36 a |
| SC | Control | 72.22±22.77 a | 81.53±15.8 ce | 0.647±0.11 cd | 0.577±0.04 a | 52.33±6.25 ac | 22.38±6.87 ab | 1.94±1.29 ab | 1.02±0.57 a | 39.5±3.60 a |
|  | Biochar | 77.77±29.18 a | 81.07±9.17 ce | 1.840±0.40 c | 0.392±0.08 ac | 47.11±2.77 bc | 20.63±6.80 ab | 2.30±1.21 ab | 1.27±0.93 a | 44.1±1.73 a |
| SiCL | Control | 75±17.4 a | 102.27±2.70 ad | 0.316±0.11 d | 0.217±0.08 bc | 52.98±6.21 ac | 17.51±1.45 ab | 3.02±1.56 ab | 1.70±0.54 a | 41.2±2.20 a |
|  | Biochar | 86.11±12.5 a | 119.7±14.59 ab | 0.603±0.12 d | 0.275±0.09 bc | 45.33±7.54 c | 16.2±2.42 ab | 4.26±2.28 a | 1.92±1.01 a | 41.2±3.01 a |
| Loam | Control | 72.22±17.21 a | 78.63±9.26 cf | 3.29±0.58 b | 0.443±0.19 ab | 41.86±2.89 c | 18.18±1.99 ab | 2.68±1.26 ab | 2.21±0.80 a | 37.6±2.35 a |
|  | Biochar | 63.88±26.70 a | 126.73±16.47 a | 4.606±0.72 a | 0.58±0.08 a | 39.11±5.24 c | 19.78± 1.79 ab | 2.53±1.31ab | 3.96±3.27 a | 44.2±2.85 a |

Note: Data presented are means ± standard deviations. Pairwise differences connecting letters were generated based on p-value of the interaction between soil types and treatments. Means followed by different letters indicate statistically significant differences among treatments.
