# Supplementary material for: Effect of wheat straw biochar addition on canola growth in different soils
Source: PLoS One. 2025 Nov 5;20(11):e0335220. doi: 10.1371/journal.pone.0335220 (PMC12588495; doi:10.1371/journal.pone.0335220)
Supplement: S3 Table — (DOCX) [file pone.0335220.s004.docx]

| **S3 Table. p-value from ANOVA test on canola yield parameters** | | | | |  |
| --- | --- | --- | --- | --- | --- |
| **Parameters** | **Branches** | **Flower count** | **No of pods** | **Seeds per pod** | **100 seeds weight (g)** |
| **Treatment (Trt)** | 0.469^ns^ | 0.021* | 0.846^ns^ | 0.146^ns^ | 0.021* |
| **Soil type** | 0.001** | 0.001** | 0.001** | 0.170^ns^ | 0.001** |
| **Trt*soil type** | 0.014* | 0.017* | 0.075^ns^ | 0.053^ns^ | 0.006** |

Note: trt = treatment, ns = non-significant, * = significant at α =5%, ** = significant at α = 1%, *** = significant at α = 10%.
