# Supplementary material for: Effect of wheat straw biochar addition on canola growth in different soils
Source: PLoS One. 2025 Nov 5;20(11):e0335220. doi: 10.1371/journal.pone.0335220 (PMC12588495; doi:10.1371/journal.pone.0335220)
Supplement: S4 Table — (DOCX) [file pone.0335220.s005.docx]

| **S4 Table. Effect of biochar and soil type on canola yield parameters** | | | | |  |  |
| --- | --- | --- | --- | --- | --- | --- |
| **Treatments** | | **Branches** | **Flower count** | **Pods** | **Seeds per pod** | **100 seeds weight (g)** |
| **LS** | Control | 8.66±2.73 ab | 182.33±88.15 ac | 55.66±11.39 ab | 22.66±4.41 ab | 0.426±0.11 ad |
|  | Biochar | 7.66±1.86 ab | 132.5±21.73 ac | 57.66±28.44 ab | 22.66±3.07 ab | 0.400±0.05 ad |
| **SL** | Control | 8.33±1.50 a | 128.83±92.04 ac | 30.33±12.70 b | 20.66±8.50 ab | 0.490±0.05 ab |
|  | Biochar | 7.16±2.14 ab | 149.5±26.18 ac | 27±9.29 b | 20.16±5.56 ab | 0.440±0.05 ad |
| **SiL** | Control | 6±1.41 ab | 119.7±94.21 bc | 37.5±16.03 b | 17.5±3.56 ab | 0.404±0.03 ad |
|  | Biochar | 12.5±4.81 a | 205.5±75.83 ac | 50±20.94 b | 22.16±4.53 ab | 0.462±0.08 ac |
| **SCL** | Control | 7.66±1.96 ab | 146.5±99.95 ac | 50.33±15.88 ab | 19.16±4.07 ab | 0.391±0.06 ad |
|  | Biochar | 8.33±5.82 ab | 241.5±108.97ab | 32.66±13.90 ab | 22±4.28 ab | 0.476±0.12 ac |
| **CL** | Control | 8.16±2.22 ab | 146.5±87.77 ac | 37.5±22.39 ab | 14.83±2.85 b | 0.414±0.09 ad |
|  | Biochar | 6.33±1.21 ab | 106.5±19.99 c | 50±12.03 ab | 23.5±3.14 ab | 0.378±0.04 ad |
| **SC** | Control | 8.83±2.13 ab | 182.33±29.50 ac | 39.83±16.04 ab | 21.83±7.31 ab | 0.426±0.09 ad |
|  | Biochar | 9.83±2.99 ab | 211.66±65.60 ac | 32.83±15.60 ab | 18.16±3.86 ab | 0.504±0.06 a |
| **SiCL** | Control | 7.83±1.83 ab | 151.16±27.87 ac | 67±9.09 a | 20.83±3.18 ab | 0.297±0.05 d |
|  | Biochar | 10.16±2.48 ab | 259.83±143.38 a | 49.33±25.77 ab | 19.16±2.48 ab | 0.473±0.12 ac |
| **Loam** | Control | 3.33±0.81 b | 90.83±10.99 c | 47.33±8.71 ab | 23.66±5.04 ab | 0.334±0.06 cd |
|  | Biochar | 4.16±2.63 b | 107.33±45.48 c | 50.83±21.58 ab | 24.5±4.32 a | 0.342±0.03 bd |

Note: Data presented are means ± standard deviations. Pairwise differences connecting letters were generated based on p-value of the interaction between soil types and treatments. Means followed by different letters indicate statistically significant differences among treatments.
